# Clinical characteristics and therapeutic strategy of granulomatous mastitis accompanied by *Corynebacterium kroppenstedtii:* a retrospective cohort study

**DOI:** 10.1186/s12905-023-02509-7

**Published:** 2023-07-25

**Authors:** ShunBo Li, Qian Huang, PeiPei Song, XiaoRong Han, ZeYu Liu, Lin Zhou, Ping Ning

**Affiliations:** 1 Department of Breast, Chengdu Women’s and Children’s Central Hospital, School of Medicine, University of Electronic Science and Technology of China, No.1617, Riyue Avenue 1St Section, Chengdu, Sichuan 611731 China; 2 School of Medicine, University of Electronic Science and Technology of China, Chengdu, Sichuan 611731 China; 3 Department of Laboratory, Chengdu Women’s and Children’s Central Hospital, School of Medicine, University of Electronic Science and Technology of China, Chengdu, Sichuan 611731 China

**Keywords:** Granulomatous mastitis, *Corynebacterium kroppenstedtii*, Clinical characteristics, Therapeutic strategy

## Abstract

**Background:**

Increasing evidence has suggested that *Corynebacterium kroppenstedtii* is associated with some cases of granulomatous mastitis, mostly based on pathology or microbiology. We aimed to identify the clinical characteristics and treatment regimens for granulomatous mastitis with *Corynebacterium kroppenstedtii* infection. Understanding these clinical features is essential for patient care.

**Methods:**

We retrospectively collected data on 201 patients who were pathologically diagnosed with granulomatous mastitis and had microbiological results of either *Corynebacterium kroppenstedtii* or no bacterial growth and recorded and analysed their demographics, clinical characteristics, and clinical outcomes.

**Results:**

There were 107 patients in the CK group and 94 patients in the negative group. Sinus formation (x^2^ = 13.028, *p* = 0.000), time to complete remission at the first treatment period (Z = -3.027, *p* = 0.002), diameter of breast mass at first-time medical consultancy (Z = -2.539, *p* = 0.011) and recurrence (x^2^ = 4.953, *p* = 0.026) were statistically significant. Age (Z = -1.046, *p* = 0.295), laterality (x^2^ = 4.217, *p* = 0.121), time to presentation since the last delivery (x^2^ = 0.028, *p* = 0.868), BMI (Z = -0.947, *p* = 0.344), lactation time (Z = -1.378, *p* = 0.168), parity (x^2^ = 1.799, *p* = 0.180), gravida (Z = -0.144, *p* = 0.885), history of lactational mastitis or abscess (x^2^ = 0.115, *p* = 0.734), local trauma (x^2^ = 0.982, *p* = 0.322), hyperprolactinemia (x^2^ = 0.706, *p* = 0.401), erythema nodosum (x^2^ = 0.292, *p* = 0.589), and nipple discharge (x^2^ = 0.281, *p* = 0.596) did not demonstrate statistical significance. Regarding recurrence related to therapeutic strategy, except for surgery combined with immunosuppressants (x^2^ = 9.110, *p* = 0.003), which was statistically significant, none of the other treatment regimens reached statistical significance. The recurrence rate of patients in the CK group using rifampicin in their treatment course was 22.0% (x^2^ = 4.892, *p* = 0.027).

**Conclusions:**

Granulomatous mastitis accompanied by *Corynebacterium kroppenstedtii* more easily forms sinuses and has a higher recurrence rate. Both of the clinical characteristics may indicate that *Corynebacterium kroppenstedtii* plays an important role in the development and progression of granulomatous mastitis. Lipophilic antibiotics may be essential for granulomatous mastitis with *Corynebacterium kroppenstedtii* infection.

## Background

Granulomatous mastitis (GM) is a rare inflammatory disease of the postlactation breast that clinically mimics breast cancer [1–5]. Recently, GM has become increasingly prevalent in China [6]. Its clinical presentation can be variable, ranging from acute infective episodes such as abscess or mastitis to breast lump resembling malignancy [5, 7, 8]. Sometimes, erythema nodosum and arthritis occur as extramammary presentations of GM [9]. The aetiology of GM is still unknown, and may be due to autoimmunity, microbiological agents, smoking, oral contraceptive pills, ethnicity, α1-antitrypsin deficiency and hormonal imbalance such as hyperprolactinemia [7, 10, 11].

Recent studies have found that particular *Corynebacterium* may be involved in the pathogenesis of some cases of GM [12]. *Corynebacterium kroppenstedtii* (*C. kroppenstedtii*, CK) is a lipophilic *Corynebacterium* first described in 1998 after isolation from a sputum specimen [13]. In 2002, *C. kroppenstedtii* was first described in a retrospective case series of histologically proven granulomatous lobar mastitis in predominantly young Polynesian women, and accounted for 40% of isolates recovered from these patients [12]. Since then, *C. kroppenstedtii* has been reported in multiple case reports and case series associated with GM [14–17], typically in young parous women [18–20]. *C. kroppenstedtii* can be identified by whole genome sequencing, matrix-assisted laser desorption ionization-time-of-flight mass spectrometry (MALDI-TOF MS), and 16S rRNA sequencing. MALDI-TOF MS has largely supplanted gene sequencing due to the higher cost, greater technical complexity, and prolonged turnaround time associated with gene sequencing [21, 22]. Recently, there has been increasing evidence of an association between corynebacterial infection and a distinct pattern termed cystic neutrophilic GM, characterized by lipogranulomas consisting of clear spaces rimmed by neutrophils and surrounding granulomatous inflammation [17, 23–28]. However, nearly all of these studies focused on pathological microorganisms, and few studies analysed the clinical features and the best treatment methods of GM patients infected with *C. kroppenstedtii*. Therefore, here, we retrospectively collected all patients who were pathologically diagnosed with GM for the first time in our hospital from 2018 to 2021 and had microbiological results, completed treatment plans and achieved cure, which was defined as breast lesions completely disappearing with surgery or drugs at least one time. Their clinical signs, management, clinical course, and clinical outcomes were collected and analysed.

Our study attempted to determine the clinical characteristics and better therapeutic strategy of GM with *C. kroppenstedtii* infection, not only to deepen and complete the understanding of the clinical features of GM with *C. kroppenstedtii* infection in the literature, but also to provide direct and objective evidence of *C. kroppenstedtii* in the development and progression of GM.

## Methods

### Ethical approval

The Institutional Review Board of Chengdu Women's and Children's Central Hospital approved this study (reference number 2022(96)). The requirement for patient consent to participate in this study was waived because of the retrospective nature of the study. Information was gathered from electrical medical systems in an anonymous manner, where all authors could ensure the confidentiality of patient data. The study followed the latest version of the Helsinki Declaration.

### Study subjects

Our study included patients who were treated in the Breast Department of Chengdu Women’s and Children’s Central Hospital between January 2018 and June 2021. The follow-up time ranged from 13 to 42 months. Eligibility criteria: a) pathologically diagnosed as GM, which means the histopathologic features are noncaseating granulomas centred on lobules, with or without associated microabscesses; b) accompanied by abscess formation throughout the course of the disease; c) routine bacterial culture (culture conditions: blood agar culture-medium, 35 degrees Celsius) of pus and MALDI-TOF MS to identify if the bacteria was *C. kroppenstedtii*; d) complete remission, which was defined as no residual lesions both clinically and ultrasonically at least one time. The exclusion criteria were as follows: a) evidence of other bacteria, b) pregnancy, and c) psychiatric illness.

### Study data

The patients’ medical records were retrieved from not only outpatient but also inpatient departments. We recorded their age, laterality, gravidity, parity, BMI, mental illness, time to presentation since last delivery and breast feeding, history of lactational mastitis or abscess, local trauma, bacterial results, serum prolactin levels and clinical findings (including diameter of breast mass at first-time medical consultancy, sinus formation, erythema nodosum, and nipple discharge), management protocols (including immunosuppressants such as steroids and methotrexate, surgery, antibiotics (antituberculosis drugs rifampicin)), time to complete remission at the first treatment periods, and recurrence. All patients were assessed by physical examination, laboratory tests and ultrasonography (USG) during the follow-up period. At the same time, we collected more information of interest from our follow-up centre, such as lactation time and recurrence which was defined as the appearance of new lesions either clinically or ultrasonically after complete remission, and the new lesions were pathologically confirmed as granulomatous mastitis. The data were analysed retrospectively.

### Statistical analysis

Data are presented as medians and ranges for quantitative variables and as numbers or percentages for qualitative variables. Quantitative data between the groups were first tested for normality and compared using the Mann–Whitney U test. The chi-square test, Fisher’s exact test and Mann–Whitney U test were used to compare qualitative data. A *p* value of < 0.05 was considered statistically significant. All statistical analyses were performed using the SPSS statistical software package version 23.0.

## Results

In total, 201 patients were enrolled in our study, and they were all Chinese parous women aged 23 to 52. There were 107 patients in the CK group (defined as those with *C. kroppenstedtii* infection) and 94 patients in the negative group (defined as those with no evidence of bacteria). Most patients had normal BMI and their baby was 5 years old or younger when they were diagnosed with GM. The majority of enrolled patients had only one child and nearly half of them lactated for 7–12 months. The details of demographics and clinical characteristics, including recurrence, are presented in Table 1, but some data on lactation time were missing for 18 patients in the CK group and 16 in the negative group. Recurrence related to treatment methods is presented in Tables 2 and 3.

**Table 1:** Demographics and clinical characteristics

Characteristics	Total *N* = 201	CK (*n* = 107)	N (*n* = 94)	P
**Age in year**	31 (23–52)	31 (23–48)	32 (23–52)	0.295
**Laterality**				0.121
Left	105 (52.2%)	52 (48.6%)	53 (56.4%)	
Right	85 (42.3%)	46 (43.0%)	39 (41.5%)	
Bilateral	11 (5.5%)	9 (8.4%)	2 (2.1%)	
**Diameter (cm)**	5.5 (2.0–17.0)	6.0 (2.0–17.0)	5.0 (2.0–17.0)	0.011
**BMI (kg/m**^**2**^**)**				0.344
< 18.5	14 (7.0%)	7 (6.5%)	7 (7.4%)	
18.6–24.9	144 (71.6%)	74 (69.2%)	70 (74.5%)	
25–29.9	35 (17.4)	22 (20.6%)	13 (13.8%)	
≥ 30	8 (4.0%)	4 (3.7%)	4 (4.3%)	
**Time to presentation since the last delivery(year)**				0.868
≤ 5	181 (90.0%)	96 (89.7%)	85 (90.4%)	
> 5	20 (10.0%)	11 (10.3%)	9 (9.6%)	
**Lactation time(month)**^a^				0.168
0	11 (6.6%)	6 (6.7%)	5 (6.4%)	
1–6	45 (26.9%)	19 (21.3%)	26 (33.3%)	
7–12	80 (47.9%)	46 (51.7%)	34 (43.6%)	
13–18	23 (13.8%)	12 (13.5%)	11 (14.1%)	
19–24	6 (3.6%)	5 (5.6%)	1 (1.3%)	
> 24	2 (1.2%)	1 (1.1%)	1 (1.3%)	
missing	34 (16.9%)	18 (16.8%)	16 (17.0%)	
**Parity**				0.180
1	156 (77.6%)	87 (81.3%)	69 (73.4%)	
> 1	45 (22.4%)	20 (18.7%)	25 (26.6%)	
**Gravidity**				0.855
1	66 (32.8%)	37 (34.6%)	29 (30.9%)	
2	73 (36.3%)	36 (33.6%)	37 (39.4%)	
3	35 (17.4%)	19 (17.8%)	16 (17.0%)	
≥ 4	27 (13.4%)	15 (14.0%)	12 (12.8%)	
**Lactational mastitis**	34 (16.9%)	19 (17.8%)	15 (16.0%)	0.734
**Local trauma**	17 (8.5%)	11 (10.3%)	6 (6.4%)	0.322
**Hyperprolactinemia**	37 (18.4%)	22 (20.6%)	15 (16.0%)	0.401
**Erythema nodosum**	9 (4.5%)	4 (3.7%)	5 (5.3%)	0.589
**Sinus formation**	100 (49.8%)	66 (61.7%)	34 (36.2%)	0.000
**Nipple discharge**	15 (7.5%)	7 (6.5%)	8 (8.5%)	0.596
**Time to complete remission (month)**	5.0 (1.0–24.0)	6.0 (1.0–24.0)	4.0 (1.0–24.0)	0.002
**Recurrence**	52 (25.9%)	35 (32.7%)	17 (18.1%)	0.018

*CK* the *Corynebacterium kroppenstedtii* group

*N* the negative group

*BMI* Body mass index

^a^Data were missing for some patients

**Table 2:** Recurrence related to treatment methods

Treatment	Patients	Recurrence	CK	N	P
**IM + antibiotics + surgery**	23 (11.4%)	6 (26.1%)	4/19 (21.1%)	2/4 (50.0%)	0.270
**IM + surgery**	107 (53.2%)	30 (28.0%)	18/40 (45.0%)	12/67 (17.9%)	0.003
**antibiotics + surgery**	9 (4.5%)	3 (33.3%)	3/5 (60.0%)	0/4 (0.0%)	0.167
**IM**	26 (13.0%)	9 (34.6%)	6/17 (35.3%)	3/9 (33.3%)	1.000
**antibiotics**	9 (4.5%)	0 (0.0%)	0/5 (0.0%)	0/4 (0.0%)	-
**IM + antibiotics**	27 (13.4%)	4 (14.8%)	4/21 (19.0%)	0/6 (0.0%)	0.545

*IM* Immunosuppressants

*CK* the *Corynebacterium kroppenstedtii* group

*N* the negative group

**Table 3:** Recurrence related to antibiotics (rifampicin) in CK group

Group	Patients	Recurrence	No recurrence	P
**A**	50	11 (22.0%)	39 (78.0%)	0.027
**B**	57	24 (42.1%)	33 (57.9%)	

*CK Corynebacterium kroppenstedtii*

*A* management protocols that included rifampicin

*B* management protocols that did not include rifampicin

Table 1 shows that the median age of all patients was 31 years, with 31 years in the CK group and 32 years in the negative group (Z = -1.046, *p* = 0.295). Eleven (5.5%) patients had bilateral lesions, and the others were all unilateral (x^2^ = 4.217, *p* = 0.121). The median diameter of the mass on the first medical consultation in our study was 5.5 cm, 6.0 cm in the CK group and 5.0 cm in the negative group (Z = -2.539, *p* = 0.011). Sinus formation (x^2^ = 13.028, *p* = 0.000) and recurrence (x^2^ = 4.953, *p* = 0.026) were statistically significant, and both were more frequent in the CK group. However, time to presentation since the last delivery (x^2^ = 0.028, *p* = 0.868), BMI (Z = -0.947, *p* = 0.344), lactation time (Z = -1.378, *p* = 0.168), parity (x^2^ = 1.799, *p* = 0.180), gravida (Z = -0.144, *p* = 0.885), history of lactational mastitis or abscess (x^2^ = 0.115, *p* = 0.734), local trauma (x^2^ = 0.982, *p* = 0.322), hyperprolactinemia (x^2^ = 0.706, *p* = 0.401), erythema nodosum (x^2^ = 0.292, *p* = 0.589), and nipple discharge (x^2^ = 0.281, *p* = 0.596) did not demonstrate statistical significance. The median time to complete remission at the first treatment period in our study was 5.0 months, ranging from 1.0 to 24.0 months, 6.0 months in the CK group, and 4.0 months in the negative group (Z = -3.027, *p* = 0.002).

Table 2 shows the different treatment strategies in our study, including surgery combined with antibiotics and immunosuppressants, surgery combined with immunosuppressants, surgery combined with antibiotics, immunosuppressants combined with antibiotics, immunosuppressants alone, and antibiotics alone. More than 50% (107) of the patients underwent surgery combined with immunosuppressants, and 30 (28.0%) patients suffered from recurrence; 18 (45.0%) in the CK group and 12 (17.9%) in the negative group (x^2^ = 9.110, *p* = 0.003). None of the patients who received antibiotics alone suffered from recurrence. However, surgery combined with antibiotics and immunosuppressants (Fisher’s exact test, *p* = 0.270), surgery combined with antibiotics (Fisher’s exact test, *p* = 0.167), immunosuppressants combined with antibiotics (Fisher’s exact test, *p* = 0.545), and immunosuppressants alone (Fisher’s exact test, *p* = 1.000) were not statistically significant.

Patients in the CK group were divided into Groups A and B according to whether they used rifampicin in their treatment course, and Group A was defined as management protocols that included rifampicin, and Group B was defined as management protocols that did not include rifampicin. The recurrence rate was 22.0% in Group A and 42.1% in Group B (x^2^ = 4.892, *p* = 0.027) (Table 3).

## Discussion

*C. kroppenstedtii* is an unusual member of the genus Corynebacterium first described in 1998 as it lacks the characteristic mycolic acids in the cell envelope [18, 29–31]. The *C. kroppenstedtii* type strain has revealed a lipophilic (lipid-requiring) lifestyle and a remarkable repertoire of carbohydrate uptake and utilization systems [3, 18, 29, 32]. Breasts are made of fat and glands and are full of lipids. Sue Paviour et al. found that *Corynebacteria* were isolated from breast tissue, pus, or deep wound swabs of 24 women; the most common species isolated was the newly described *C. kroppenstedtii* [12]. The rich lipid environment of the breast is conducive to the growth and reproduction of *C. kroppenstedtii* [33–35]. Thus, the breast is a favourable environment for the development of lipophilic corynebacteria. Moreover, P. Kieffer et al. thought that mammary areas rich in lipids or malformations such as ductal ectasia can be a factor favour the development of GM [15]. Therefore, lipid-rich breast tissue provides the perfect habitat for *C. kroppenstedtii* to reside and form granulomas and abscesses [36]. According to recent studies, increasing evidence has confirmed that GM is closely related to *C. kroppenstedtii*. Yu HJ et al. confirmed the predominance of *C. kroppenstedtii* infection in GM patients (11 of 19 patients, 57.9%) with Sanger sequencing and qPCR assays [13]. Tariq et al. found that *C. kroppenstedtii* 16S rRNA real-time polymerase chain reaction was positive on formalin-fixed, paraffin-embedded tissues from 46 of 67 (68.7%) GM cases [37]. Li XQ et al. achieved a detection rate of *C. kroppenstedtii* up to 56% in the nanopore sequencing method [38]. In our study, using MALDI-TOF MS, *C. kroppenstedtii* was confirmed in more than 50% of patients as well.

From our study, 61.7% of patients in the CK group formed sinuses, compared to the negative group, and patients in the CK group seemed to be more likely to form sinuses, which means that sinus formation was more common in GM with *C. kroppenstedtii* in our study than in those with no evidence of bacteria. Taylor et al. conducted research on a case group (34 patients with *Corynebacterium* spp.) versus the control group (28 patients with no evidence of Corynebacteria) and found that the formation of draining sinuses was significantly different between the two groups and was more frequent in the case group [1]. Bi JX et al. found that 16 of 25 (64.00%) patients diagnosed with GM in their study had skin ulceration and pus, and 9 of them (56.25%) with skin ulceration had the pathogen *C. kroppenstedtii* [33]. *C. kroppenstedtii* preferred to grow and proliferate in lipid-rich areas of the breast, including the subcutaneous area, first forming small and scattered localized abscesses and then gradually expanding and infiltrating the subcutaneous area and forming sinuses. Thus, it is much easier for GM to form sinuses with *C. kroppenstedtii* infection.

Except for sinus formation, we also found that recurrence may be associated with *C. kroppenstedtii*. In our study, a total of 52 patients (25.9%) suffered from recurrence, and among them, 35 patients were found to be positive for *C. kroppenstedtii*. In the literature, there is a recurrence rate of approximately 24.8% for GM whether *C. kroppenstedtii* infection or not [39]. Some studies also reported that the presence of *C. kroppenstedtii* was a significant prognosticator for recurrence [5, 40]. Azizi A et al. found that breast skin lesions were associated with significantly higher odds of recurrence, but unfortunately, there was no further exploration with *C. kroppenstedtii* [39]. In conclusion, GM accompanied by *C. kroppenstedtii* is much easier to recur. Given the high rate of recurrence, close long-term follow-up must be emphasized [41].

According to the literature, the most common treatments used in *C. kroppenstedtii* breast infections are surgery, steroids, and antibiotics, but their individual and combined impact is unclear [2, 36, 42]. In addition, close observation might be the optional management for GM [42–44]. Godazandeh carried out a meta-analysis and reported that the combination of steroids and surgery was more effective than steroids only [45]. Additionally, most Chinese experts agreed that corticosteroid combined surgery was used as the primary treatment for GM [46, 47]. Methotrexate is a treatment option for patients who have relapsed or who do not tolerate high-dose corticosteroid therapy [28, 48]. Surgery was performed depending on the individual clinical efficacy and the choice of surgical technique varied from wide excision to mastectomy even with transverse rectus abdominis musculocutaneous (TRAM) flaps, in the literature [28, 41, 49]. Hazel C. Dobinson et al. considered that if granulomatous disease is present, it seems prudent to choose agents that are both active against Corynebacterium spp. and have physicochemical properties that would promote activity within the lipid-filled spaces. Preferred choices include clarithromycin and rifampicin, which are also active in other granulomatous infections such as mycobacterial infections [2]. In our study, we tried multiple treatment strategies including surgery combined with antibiotics and immunosuppressants, surgery combined with immunosuppressants, surgery combined with antibiotics, immunosuppressants combined with antibiotics, immunosuppressants alone, and antibiotics alone according to the individual clinical appraisal. Most of patients underwent surgery combined with immunosuppressants, and 30/107(28.0%) of them who accepted this treatment regimen suffered relapses, and patients with *C. kroppenstedtii* had a higher rate of recurrence (45%), but the rate of recurrence in those patients with *C. kroppenstedtii* who were treated with lipophilic antibiotics combined or alone was only 22% (11/50). Thus, lipophilic antibiotics may be essential for GM with *C. kroppenstedtii* infection when formulating treatment protocols, but more evidence and prospective studies are needed.

Davis et al. reported that the average time to resolution was 5 months (range 0–20) in their 120 patients identified with GM [43]. In our study, the median time to complete remission was 5.0 months as well, ranging from 1.0 to 24.0 months, and patients in the CK group needed more time to achieve cure at their first treatment periods than those in the negative group. However, as reported in the literature, surgery that could easily change the time of achieving cure at their first treatment periods was performed not only depending on clinicians’ judgement but also taking into account the patients’ wishes [28]. In our study, nearly 70% of patients underwent surgery. Therefore, although the time to complete remission in our study was different, we still need to be cautious. The diameter of the mass on the first medical consultation in our study was not completely consistent because the mass of GM grows and spreads rapidly [50], and the time of their first medical consultation after the initial presentation of the mass was not completely consistent.

To the best of our knowledge, this is the largest study in the literature discussing the clinical characteristics and therapeutic strategy of GM with *C. kroppenstedtii* infection. However, we still have some limitations of the current study. First was its retrospective nature, and missing some data of partial patients about the lactation time. Second, except for surgery combined with immunosuppressants there were too few patients treated with other protocols. Third, it is difficult to grow CK in the laboratory; therefore, those with "no CK" isolated might in fact be CK that just did not grow. In brief, our findings should be interpreted prudently and validated in future studies.

## Conclusions

Sinus formation and a higher recurrence rate are intimately related to *C. kroppenstedtii* infection, and both may prove the possible important role of *C. kroppenstedtii* in the development and progression of GM. Although the time to complete remission at the first treatment period and the diameter of the mass at the first medical consultation were significantly different in our study, we still need to be cautious. In addition, lipophilic antibiotics may be considered for GM with *C. kroppenstedtii* infection when developing treatment plans.

## Data Availability

All data generated or analyzed during this study are included in this published article/as supplementary information files.

## Abbreviations

GM: Granulomatous mastitis
CK: *Corynebacterium* *kroppenstedtii*
MALDI-TOF MS: Matrix-assisted laser desorption ionization-time-of-flight mass spectrometry
USG: Ultrasonography
TRAM: Transverse rectus abdominis musculocutaneous
